# circ_0000567/miR-421/*TMEM100* Axis Promotes the Migration and Invasion of Lung Adenocarcinoma and Is Associated with Prognosis

**DOI:** 10.7150/jca.60124

**Published:** 2022-02-28

**Authors:** Yang Hong, Jiahui Si, Bufan Xiao, Ying Xiong, Chenyue Dai, Yue Yang, Shaolei Li, Yuanyuan Ma

**Affiliations:** Department of Thoracic Surgery II, Key Laboratory of Carcinogenesis and Translational Research (Ministry of Education), Peking University Cancer Hospital and Institute, Beijing, People's Republic of China

**Keywords:** LUAD, Hsa-miR-421, Hsa_circ_0000567, TMEM100, prognosis

## Abstract

**Purpose:** Due to the high metastatic ability and poor prognosis of lung adenocarcinoma (LUAD), we identified novel non-coding RNAs, which constitute approximately 60% of human transcripts, as prognostic biomarkers and potential therapeutic targets for LUAD.

**Methods:** In this study, we downloaded and analyzed microRNA (miRNA) datasets from The Cancer Genome Atlas (TCGA) to identify dysregulated miRNAs correlating with the overall survival (OS) of LUAD patients. miR-421, circ_0000567, and *TMEM100* expression levels were examined by quantitative real-time polymerase chain reaction (qRT-PCR) in NSCLC tissues from 73 patients and adjacent normal tissues. Cell migration and invasion were assayed using wound healing and transwell assays. miR-421 target predictions were conducted using starBase, CircInteractome, circBank, TargetScan, miRanda, MirDB, miRpath, and Gene Expression Omnibus (GEO) databases. The circular structure and stability of circ_0000567 were verified by RNase R digestion and qRT-PCR using oligo(dT) and random primers. A luciferase reporter assay was used to evaluate the relationship between miR-421, circ_0000567, and *TMEM100.*

**Results:** The miRNA panel associated with OS in patients with LUAD was screened according to the hazard ratio (HR) of miRNAs from high to low. Based on the correlation between these miRNAs and OS, as well as miRNA expression levels, miR-421 was selected for further outcome analysis. High miR-421 expression was an independent risk factor for shorter OS in 73 patients collected from our department. Bioinformatic analyses, luciferase reporter assays, and functional assays showed that circ_0000567 could act as a sponge for miR-421 and prevent it from directly targeting the 3'-untranslated region of TMEM100 mRNA and further degrading it in LUAD. miR-421 expression was significantly upregulated, while circ_0000567 and *TMEM100* were downregulated in tumor tissues of LUAD, compared to their counterparts in normal tissues. Gain- and loss-of-function assays showed that miR-421 promoted LUAD cell migration and invasion. Overexpression of circ_0000567 inhibited migration and invasion, whereas co-transfection of circ_0000567 and miR-421 mimics partly counteracted this effect. *TMEM100* was upregulated by enhanced circ_0000567 in LUAD cells, and the expression of *TMEM100* was inversely proportional to miR-421, whereas it was directly proportional to circ_0000567 in 73 LUAD specimens, which confirmed the competitive endogenous RNA (ceRNA) network.

**Conclusion:** Our findings suggest that miR-421 promotes the migration and invasion of lung adenocarcinoma via circ_0000567/miR-421/*TMEM100* signaling and could be a prognostic biomarker for LUAD.

## Introduction

Lung cancer is one of the leading causes of cancer-related deaths worldwide^1^. It is estimated that the number of lung cancer deaths will be 3 million in 2035^2^, ^3^. Non-small cell lung cancer (NSCLC) accounts for approximately 85% of lung cancer cases, with lung adenocarcinoma (LUAD) being the major pathological types 4-6. With decreasing smoking rates, non-smoking associated lung cancer are increasing, most of which comprise LUAD 7.Therefore, the pathogenesis, characteristics, and treatment of LUAD have recently drawn significant attention.

It is reported that over 90% of the human genome is actively transcribed; however, the human genome encodes only approximately 20,000 protein-coding genes, accounting for less than 2% of the genome sequence. Functional non-coding RNAs (ncRNAs), including microRNAs (miRNAs), long non-coding RNAs (lncRNAs), pseudogenes, and circular RNAs (circRNAs), constituting almost 60% of the transcriptional output in humans, have recently attracted considerable attention in cancer research^8^-^12^.The complex regulatory networks between ncRNAs and protein-coding genes suggest that ncRNAs play key roles in tumor proliferation, metastasis, and drug resistance 9, 13. Their aberrant expression, tissue specificity, complex regulation network, high transcription level, and stability in paraffin-embedded tissues and body fluids, even in plasma and serum, make ncRNAs a good biomarker in many cancers 13, 14. While most previous studies focused on ncRNAs as diagnostic markers, recent studies have found that ncRNAs can act as prognostic markers and may have potential as therapeutic targets in LUAD 15-17.

One miRNA can target many genes according to their binding sites, and one gene can be targeted by many different miRNAs, leading to complex post-transcriptional inhibition. According to the competitive endogenous RNAs (ceRNAs) hypothesis, circRNAs or lncRNAs compete for the same group of miRNA-responsive elements (MRE) with target mRNAs, which in turn affect tumor progression 18, 19. circRNAs are very stable due to their specific loop structure without 3′ and 5′ ends, and therefore, they are resistant to exonuclease activity 11. Hence, circRNAs have received significant attention in the past decade. They have been confirmed to be related to cell proliferation, invasion, migration, and apoptosis in previous LUAD studies 20-31.

ncRNAs with different expression levels in tumors and corresponding normal tissues have been previously selected for further clinical studies^32^. However, we performed our study from another angle. Based on datasets downloaded from The Cancer Genome Atlas (TCGA), we screened candidate ncRNAs by evaluating the likelihood of correlation between ncRNAs and overall survival (OS) in patients with LUAD. We started our study by focusing on the most widely studied ncRNAs, microRNAs, at first.We conducted an integrated analysis of LUAD-specific data derived from TCGA and found an OS-related microRNA (miR-421) in LUAD. Next, the expression level of miR-421 and the role of miR-421 in LUAD cell lines were further analyzed. Our results revealed that miR-421 could be sponged by circ_0000567 and directly target *TMEM100* mRNA. We are trying to provide new prognostic biomarkers and suggest novel directions for the improvement of prognostic prediction and therapeutic processes for LUAD in the clinic.

## Materials and Methods

### Tissue specimens

Eligible criteria for patient recruitment included: (1) histological confirmed LUAD; (2) no neoadjuvant therapy; (3) performed R0 resection;(4) complete basic information, including age, gender, histology, TNM stage, and follow-up data; (5) no diagnosis of any other severe diseases. The exclusion criteria were: (1) patients received radiotherapy or chemotherapy before surgery; (2) a history of malignancies other than LUAD within 5 years. Based on these criteria, LUAD tissues and matched adjacent normal lung tissues were collected from patients with LUAD who underwent surgery at Peking University Cancer Hospital (Beijing, China) from December 2011 to December 2012. Paraffin tumor tissues from 73 patients with LUAD who had undergone surgery were investigated. TNM stage classification complied with the TNM classification system of the NCCN guidelines (Table 1). All participants provided written informed consent, and the study was supervised by the Ethics Committee of Peking University Cancer Hospital. The study was conducted in accordance with the principles of the Declaration of Helsinki.

### Cell culture

Human LUAD cell lines (A549, PC9, H1299) and human normal lung epithelial cells (BEAS-2B) were maintained in our laboratory. All cell lines were cultured in RPMI 1640 medium (Gibco BRL, Gaithersburg, MD), supplemented with 10% fetal bovine serum (FBS), 100 U/mL penicillin, and 100 g/mL streptomycin (Invitrogen, Carlsbad, CA), and were cultured in a humidified atmosphere containing 5% CO_2_ at 37°C.

### Quantitative real-time PCR (qRT-PCR)

Total RNA was extracted using TRIzol reagent (Invitrogen) according to the manufacturer's protocol. Reverse transcription of 2 μg total RNA was performed using the EasyScript First-Strand cDNA Synthesis SuperMix (Transgen Biotech, Beijing, China). For the microRNA analysis, a poly(A) tail was added to total RNA (0.5 μg) by *Escherichia coli* poly(A) Polymerase (Invitrogen), oligo(dT) adapter primers, and M-MLV Reverse Transcriptase (Invitrogen) for reverse transcription. RT-qPCR was performed using the 7500 Fast Real-Time PCR System (ThermoFisher Scientific, Waltham, MA) and Go Taq qPCR Master Mix (Promega, Madison, WI). The cycling conditions for RT‑qPCR were as follows: 95°C for 5 min, followed by 45 cycles of 95°C for 10 s, 55°C for 30 s, and 72°C for 30 s. Relative expression levels of miR-421, circ_0000567, and *TMEM100* were normalized to those of endogenous U6, GAPDH, and β-actin, respectively. The fold changes in transcript levels were calculated using the 2^-ΔCt^ method. All primers are listed in Table S1.

### Circular structure confirmation

Total RNA (5 μg) extracted from A549 cells was incubated at 37°C for 5 min with or without 5 U/ug RNase R (Epicentre Technologies, Madison, WI). After treatment, the expression levels of SETD3 and circSETD3 were determined by qRT-PCR at the same time using convergent and divergent primers separately. In addition, we also use two kinds of primers, random and oligo (dT) to perform reverse transcription in same two sets of total RNA separately, and then detected cric_0000567 and SETD3 using divergent and convergent primers separately by qPCR.

### Vector construction and transfection

The hsa_circ_0000567-pLCDH_ciR-overexpressing plasmid was synthesized by Geneseed (Guangzhou, China). hsa-miR-421 mimics, mimic negative control (NC), hsa-miR-421 inhibitor, and inhibitor NC were synthesized and purchased from Ribobio (Guangzhoou, China).

### Transwell assays

Transwell insert chamber plates were used for migration and invasion assays. Transfected cells (1-3× 10^5^) in 200 µL of RPMI 1640 medium supplemented with 1% FBS was added to the upper chamber and incubated at 37°C for 6-24 h. The lower chamber was filled with 1 mL RPMI 1640 medium supplemented with 10% FBS, and the upper chamber was coated with 60 µL diluted Matrigel (1:10, Corning Inc., Corning, NY) for the invasion assay. Non-migratory and non-invaded cells in the upper chamber were scraped off using cotton swabs. The migratory and invading cells were fixed in 4% paraformaldehyde and stained with 0.1% crystal violet. Cells were then photographed under a microscope (100× magnification) in three fields randomly, and then analyzed using Image J software (National Institutes of Health, Bethesda, MD).

### Wound healing assays

To image the same position every time, we marked the six-well plates with six straight lines in advance. PC9 and A549 cells were seeded into 6-well plates and transfected with miR-421 mimics, mimic NC, miR-421 inhibitors, or inhibitor NC.After transfection for 24 h, we performed wound healing assays. Cells were scraped with the tips of 200-μL pipette tips along the center of each well. After washing three times with PBS, the culture medium was changed to RPMI 1640 medium without FBS to eliminate the effects of proliferation. Subsequently, we used four low-power fields to photograph at 0, 6, 12, 24, and 48 h after injury.

### CCK8 assays

PC9 and A549 cells were seeded in 96-well plates at a density of 3×10^3^ cells/well, and later transfected miR-421 mimics, mimic NC, miR-421 inhibitors, or inhibitor NC. After 24 h, the number of viable cells was assessed by optical density examined at 450 nm using a CCK8 assay (Dojindo, Kumamoto, Japan) at 0, 24, 48, 72, and 96 h according to the manufacturer's instructions.

### Luciferase reporter assays

Binding sites of miRNA, circRNA, and mRNA were predicted using the starBase database. We designed wild-type and mutant 3'-untranslated regions (UTRs) of predicted mRNAs and circRNAs, synthesized these different fragment sequences, and then inserted them into the luciferase vector (Ribobio). All vectors were verified by sequencing. Then, 500 ng of wild-type or mutant plasmids were co-transfected with 50 nM miR-421 mimics or mimic NC into PC9 cells in 24-well plates. Firefly and Renilla luciferase activities were measured using the Dual-Glo® Luciferase Assay System (E2920, Promega).

### Statistical analysis

All experiments were performed in triplicate. Continuous data were expressed as mean ± standard deviation and were compared using Student's t-test as appropriate. Categorical variables were presented as counts. Correlations between miR-421 expression and clinical variables were analyzed using the 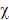
2-test. Logistic proportional hazards regression analysis was used to analyze the multivariate hazard ratios of miR-421 expression. The Kaplan-Meier method and the log-rank test were used to estimate the OS of the enrolled patients. Multivariate survival analysis was performed using a Cox proportional hazard model.All statistical analyses were performed using SPSS (version 23.0; IBM, Armonk, NY). Statistical significance was set at P<0.05.

## Results

### A panel of miRNAs was correlated with OS in LUAD

**Figure 1:**
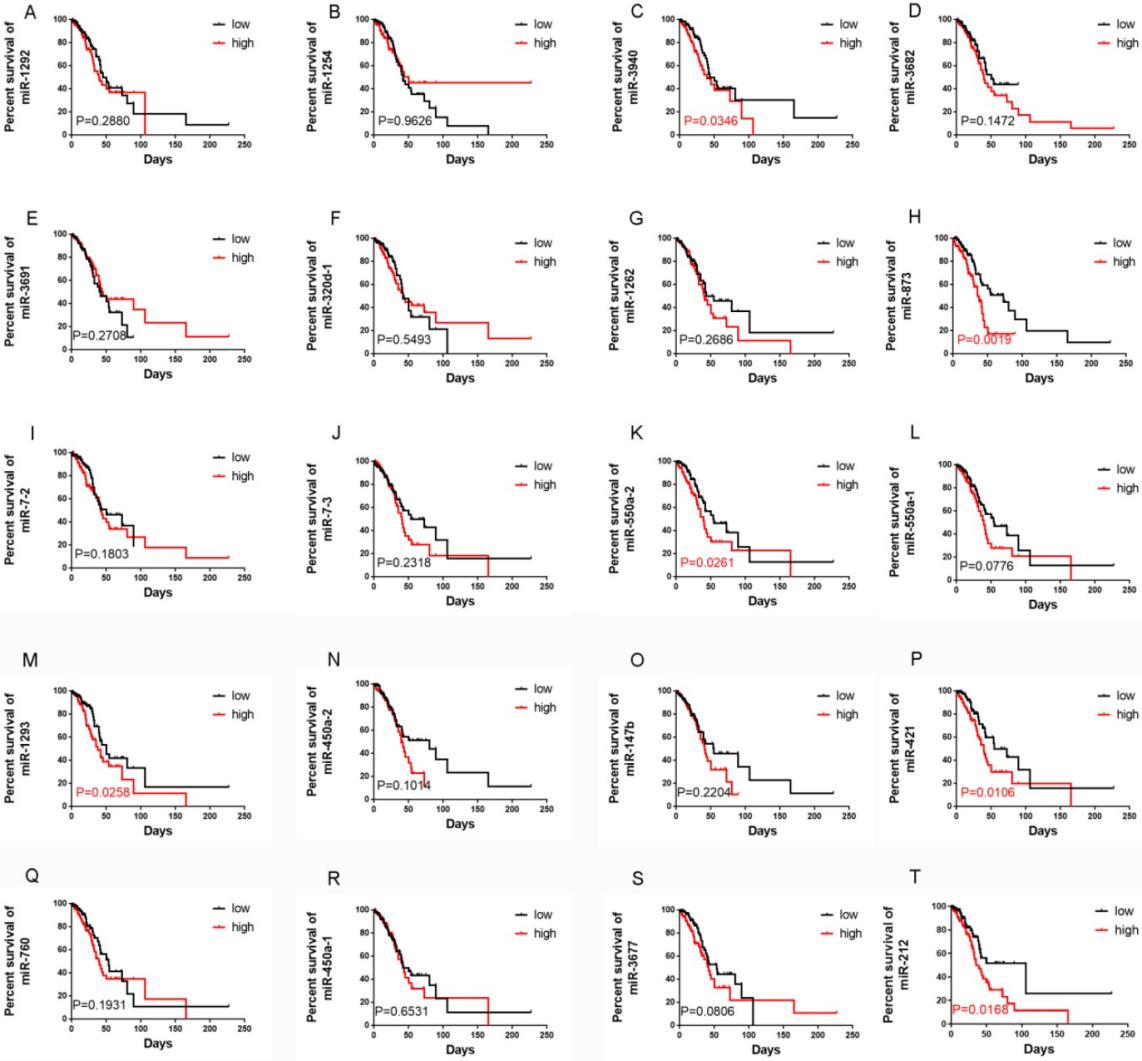
Correlations between the expression levels of 20 miRNAs, which were selected according to Cox survival analyses of 240 lung adenocarcinoma patients' data from The Cancer Genome Atlas, and overall survival (OS), respectively. Expression levels of miR-1292 **(A)**, miR-1254 **(B)**, miR-3682 **(D)**, miR-3691 **(E)**, miR-320d-1 **(F)**, miR-1262 **(G)**, miR-7-2 **(I)**, miR-7-3 **(J)**, miR-550a-1 **(L)**, miR-450a-2 **(N)**, miR-147b **(O)**, miR-760 **(Q)**, miR-450a-1 **(R)**, and miR-3677 **(S)** were not related to OS. Patients with high miR-3940 **(C)**, miR-873 **(H)**, miR-550a-2 **(K)**, miR-1293 **(M)**, miR-421 **(P)**, and miR-212 **(T)** expression had shorter OS (P<0.05).

A total of 240 LUAD RNA-seq datasets with corresponding clinical data were downloaded from TCGA (https://gdc-portal.nci.nih.gov/).We found that 61 miRNAs were significantly correlated with OS using univariate Cox regression analysis, and the mean relative expression levels of these miRNAs ranged from 0.53 to 18419.60, indicating that the mean expression level of all 61 miRNAs was >0.5 (**Table 1**). Next, we chose the top 20 miRNAs according to their EXP (coef) rankings from high to low. We then performed Kaplan-Meier analysis to confirm the correlation between miRNA expression levels and OS (**Figure 1**). Patients with higher expression levels of miR-3940, miR-873, miR-550a-2, miR-1293, miR-421, and miR-212 had a shorter OS than those with lower expression levels. In addition, analysis using the KM plotter website (http://kmplot.com/analysis/) of 504 LUAD patients revealed similar results, where the expression levels of miR-3940, miR-873, miR-550a-2, miR-1293, miR-421, and miR-212 were correlated with OS in LUAD (**Figure S1**). The relative expression levels of these miRNAs according to the TCGA datasets were shown in **Figure S2**. miR-421 and miR-212 were the most highly expressed among these six miRNAs. Finally, we examined the influence and molecular mechanism of miR-421 in LUAD, since miR-421 has rarely been reported in relation to LUAD.

### miR-421 was up-regulated in LUAD cell lines and tissues

**Figure 2:**
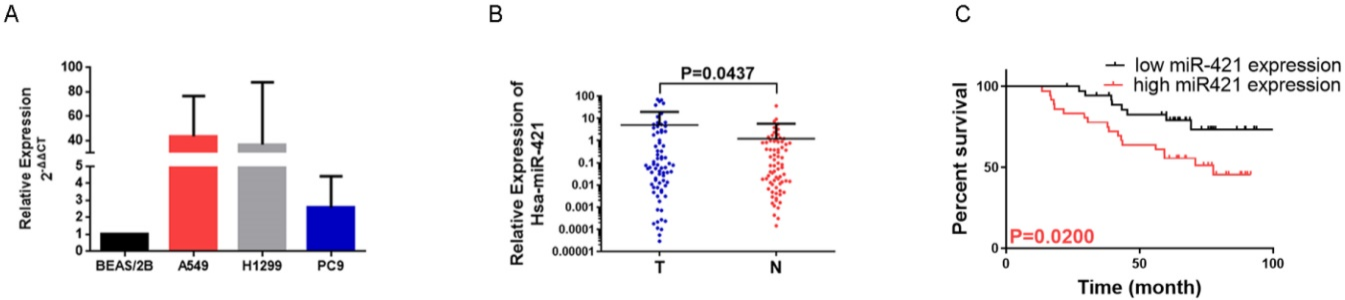
miRNA-421 expression in lung adenocarcinoma (LUAD). **A** miR-421 was upregulated in LUAD cell lines, including A549, PC9 and H1299 cells, compared to the control BEAS/2B cells. **B** miR-421 was upregulated in 73 LUAD specimens compared to paired normal lung tissues (P=0.0437). **C** LUAD patients with high levels of miR-421 had a shorter overall survival; compared to those with low expression (P=0.0200).

To confirm the role of miR-421 in LUAD tumorigenesis and metastasis, we verified the expression of miR-421 in three LUAD cell lines and a BEAS/2B control cell line using RT-qPCR. miR-421 was highly expressed in LUAD cell lines, including A549, H1299, and PC9, compared to the normal alveolar epithelial cell line (BEAS/2B). miR-421 expression was the lowest in PC9 cells and the highest in A549 cells (**Figure 2A**). Furthermore, we also examined the relative transcript levels of miR-421 in 73 LUAD tissues and paired normal lung tissues obtained from the specimen repository of our hospital using qRT-PCR. miR-421 was found to be highly expressed in LUAD specimens compared to that in paired normal tissues (**Figure 2B**).

### miR-421 is an independent risk factor for prognosis

Increased miR-421 expression was significantly associated with sex (P=0.0468), smoking habits (P=0.0454), differentiation (P=0.012), and 5-year survival (P=0.0114) (**Table 2**). In addition, when comparing the low miR-421 expression and high miR-421 expression groups, the survival analysis using the Kaplan-Meier survival curve of these 73 patients showed that patients with higher expression levels of miR-421 had a shorter OS than those with lower expression levels (P=0.0200) (**Figure 2C**). Cox multivariate survival analysis revealed that high miR-421 expression was an independent prognostic factor for poor survival in LUAD patients (hazard ratio [HR] = 2.593, 95% confidence interval [CI] 1.127-5.966, P=0.025) (**Table 3**). These data demonstrate the oncogenic role of miR-421 in LUAD.

### miR-421 promoted the invasion and migration of LUAD cell lines

**Figure 3:**
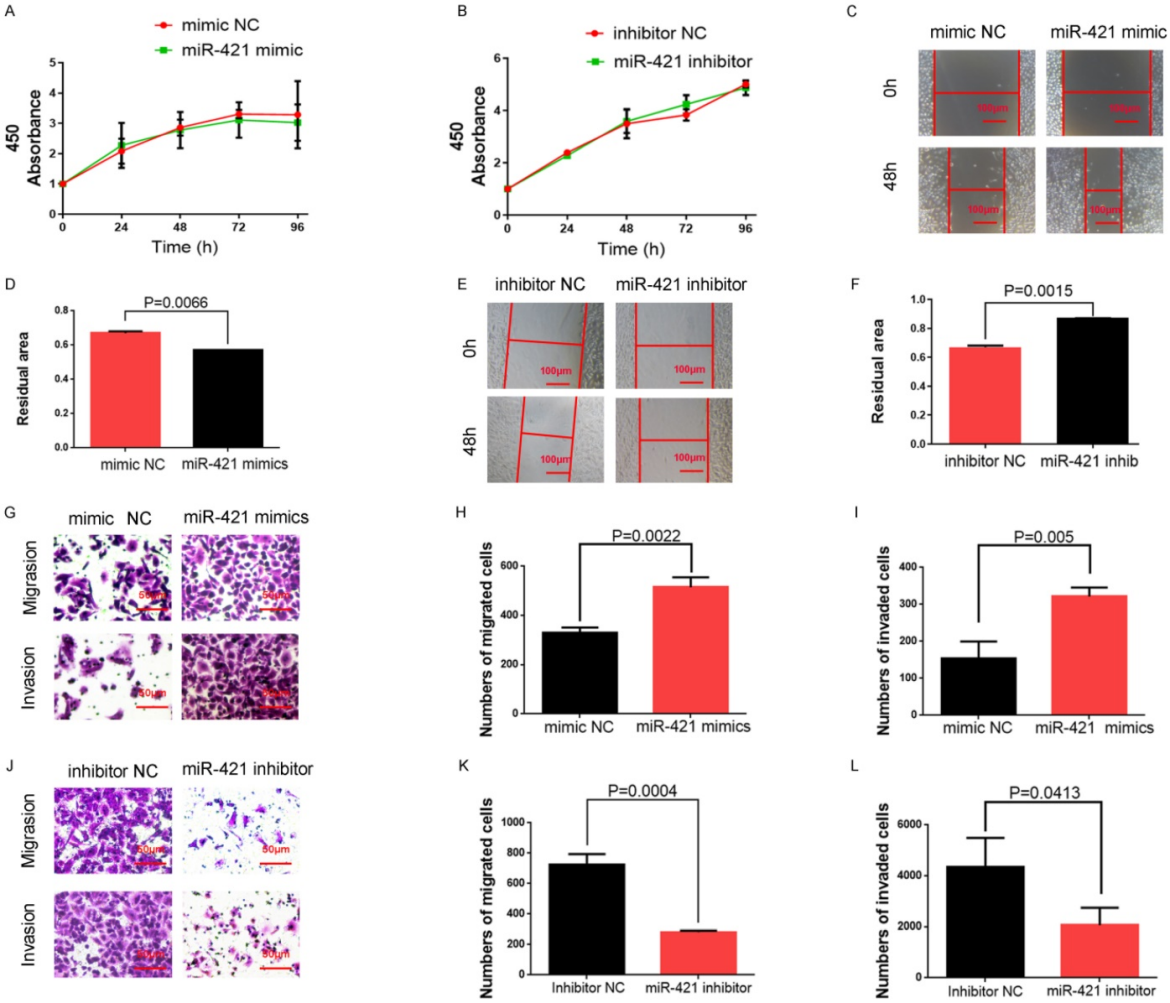
miR-421 promoted migration and invasion in lung adenocarcinoma (LUAD) cell lines. **A-B** The CCK8 assay showed that miR-421 mimics and inhibitors did not influence the cell proliferation ability of PC9 and A549 cells. **C-D** Wound healing assays showed that miR-421 mimics promoted PC9 cell migration. **E-F** Wound healing assays showed that miR-421 inhibitors decreased A549 cell migration. **G-I** Transwell assays showed that miR-421 mimics promoted PC9 cell migration and invasion.** J-L** Transwell assays showed that miR-421 inhibitors decreased A549 cell migration and invasion.

We selected A549 cells with high miR-421 expression levels for miR-421 RNA interference analysis using miR-421 inhibitors (Ribobio), and PC9 cells with low miR-421 expression levels were selected for miR-421 overexpression analysis using miR-421 mimics (Ribobio) (**Figure 2A**). We didn't find any significant differences of proliferation ability neither when LUAD cell lines transfected with miR-421 mimics nor when transfected with inhibitors, using a CCK8 assay (**Figure 3A-B**). The wound healing assay showed that the migratory ability of LUAD cells was enhanced when they were transfected with miR-421 mimics (**Figure 3C-D**), and this ability decreased when the cells were transfected with miR-421 inhibitors (**Figure 3E-F**). The migratory and invasive abilities of the LUAD cell lines were further confirmed using a transwell assay, which showed that, compared to the control groups, these abilities increased when PC9 cells were transfected with miR-421 mimics (**Figure 3G-I**) and decreased when A549 cells were transfected with miR-421 inhibitors (**Figure 3J-L**).

### miR-421 was sponged by hsa_circ_0000567 in LUAD

**Figure 4:**
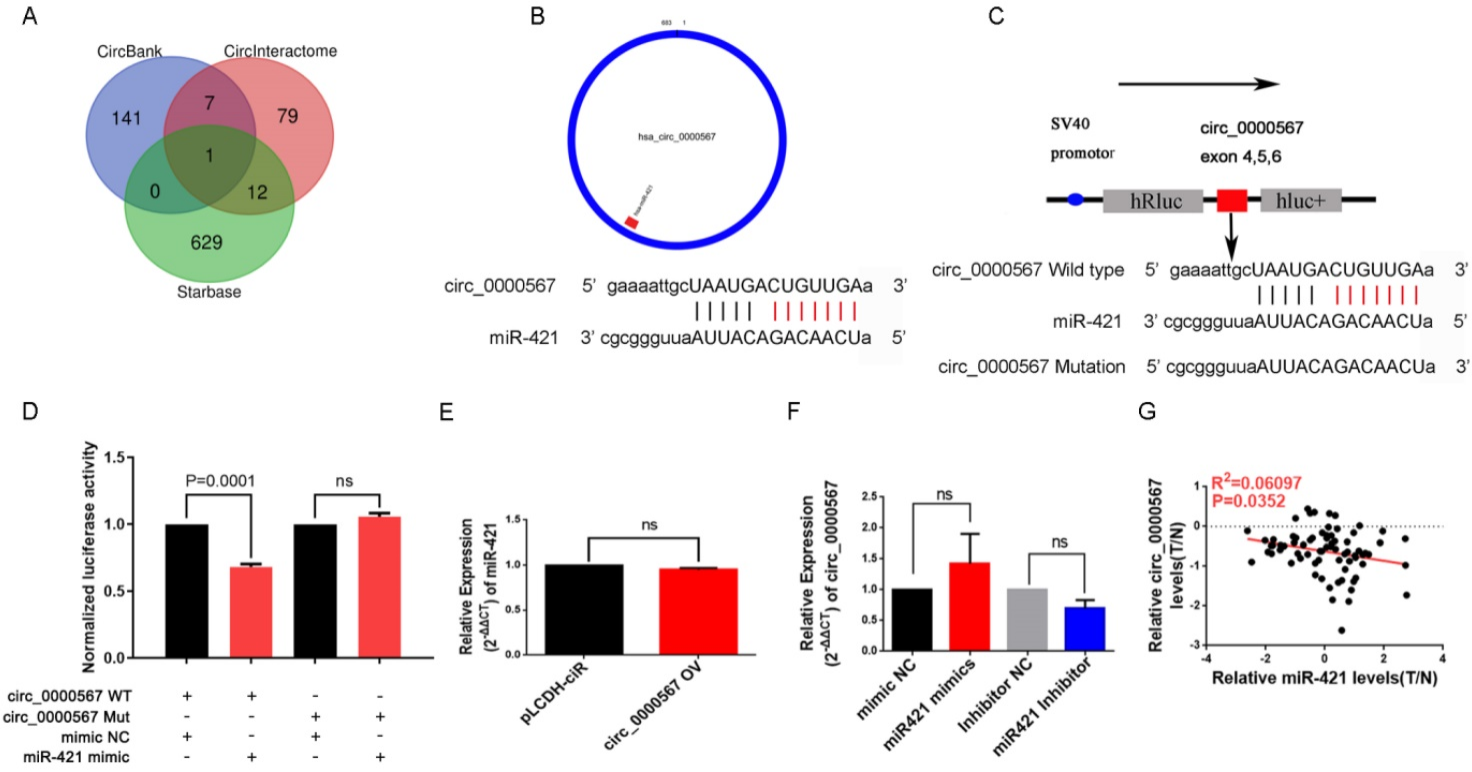
miR-421 was sponged by circ_0000567. **A** circ_0000567 was predicted as a miR-421 sponge by CircInteractome, circBank, and starBase. **B** The predicted binding site between miR-421 and circ_0000567. **C** Schematic of circ_0000567 wild-type (WT) and mutant luciferase reporter vectors. **D** Luciferase activity of WT and mutant circ_0000567 after co-transfection with miR-421 mimics. **E** Expression change of circ_0000567 did not affect miR-421 expression in LUAD cells. **F** Expression change of miR-421 did not affect circ_0000567 expression in LUAD cells. **G** miR-421 expression was negatively correlated with circ_0000567 expression.

Many miRNAs are known to be sponged and suppressed by circRNAs. Thus, we utilized the starBase, CircInteractome, and circBank databases to identify circRNAs with possible binding sites for miR-421 (**Figure 4A**). Coincidentally, only circ_0000567 was identified by all three databases and had the highest binding power score. circ_0000567 is derived from cyclizing five exons (exon 2, 3, 4, 5 and 6) from the *SETD3* gene, located at 14q32.2, and miR-421 is predicted to bind to exon 5. A schematic diagram showing circ_0000567 sponging miR-421 at exon 5 was obtained from circPrimer software (http://www.bioinf.com.cn/) and the Bioinformatics website (http://www.bioinformatics.com.cn) (**Figure 4B**).

To verify the sponging relationship between circ_0000567 and miR-421, we constructed wild-type and mutant circ_0000567 luciferase reporters. The pmiR-RB-REPORT™ vector and the binding site (mutant site) are shown in **Figure 4C.** PC9 cells were co-transfected with wild-type or mutant circ_0000567 luciferase reporters and miR-421 or mimics NC. As illustrated in **Figure 4D**, after 48h of transfection, miR-421 notably impeded the luciferase activity of the wild-type circ_0000567 reporter, whereas it had no such influence on the luciferase activity of the mutant circ_0000567 luciferase reporter.

In addition, we found that overexpression of circ_0000567 did not change the expression level of miR-421 in A549 cells. Coincidently, transfection of miR-421 mimics or inhibitors in PC9 cells did not influence the expression level of circ_0000567 either (**Figure 4E-F**), indicating that circ_0000567 only acted as a sponge for miR-421, and that they did not influence each other's expression. Furthermore, we also analyzed the expression levels of circ_0000567 and miR-421 in 73 LUAD tissue, and found that miR-421 expression level was negatively related to circ_0000567 expression level (**Figure 4G**).

### circ_0000567 sponged miR-421 to suppress the migration and invasion of LUAD cells

**Figure 5:**
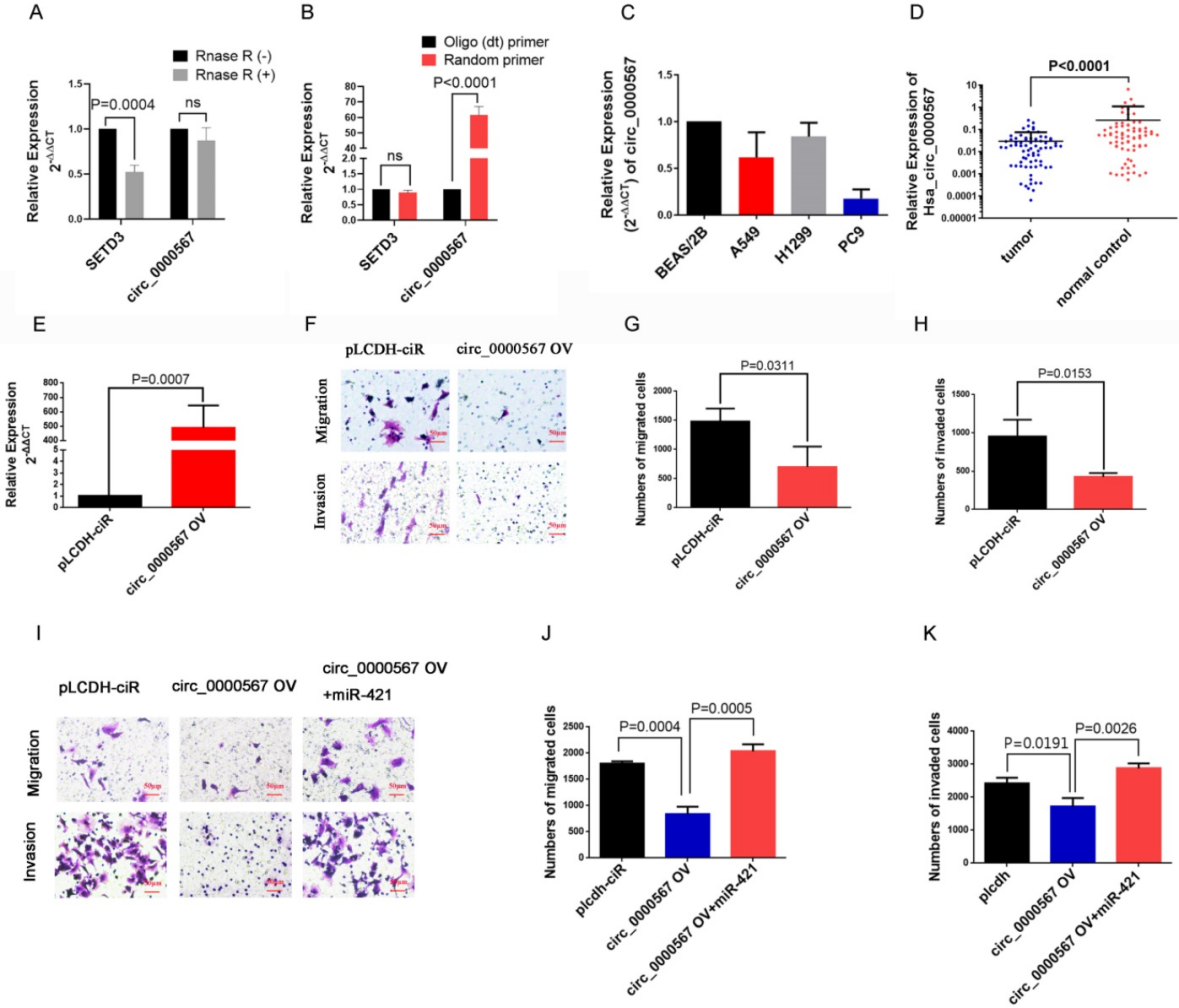
circ_0000567 suppresses migration and invasion of miR-421 by sponging miR-421. **A** circ_0000567 was resistant to RNase R, whereas the linear *SETD3* transcript could be degraded by RNase R. **B** circ_0000567 could only be reverse transcribed using random primers, but not oligo(dT) primers. However, there was no difference between using random primers or oligo(dT) primers in reverse transcription of the linear *SETD3* transcript. **C** circ_0000567 was downregulated in lung adenocarcinoma (LUAD) cell lines, including A549, PC9, and H1299, compared to control BEAS/2B cells. **D** circ_0000567 was verified to be downregulated in the 73 LUAD specimens compared to the paired normal lung tissues (P<0.0001). **E** qRT-PCR analysis of circ_0000567 expression after treatment with circ_0000567 overexpression plasmids. **F-H** circ_0000567 reduced cell migration and invasion as determined by transwell assays with and without Matrigel. **I-K** miR-421 partially abolished the effects of circ_0000567 on cell migration and invasion, as revealed by transwell assay in LUAD cells.

To confirm the circular characteristics of circ_0000567, the circular and linear transcripts of *SETD3* were examined using qRT-PCR (convergent and divergent primers separately) in A549 cell lines treated with or without RNase R. RNase R, derived from the *E. coli* RNR superfamily, cuts and degrade RNA from the 3'-5' direction and digests almost all linear RNA molecules, but does not easily digest circular, noose-shaped, or double-stranded RNA molecules with protruding ends of less than 7 nucleotides at the 3' end^33^. This RNase R digestion assay showed that the linear *SETD3* transcript could be digested by RNase R, but circ_0000567 was resistant to RNase R digestion in A549 cells (**Figure 5A**), which confirmed the loop structure of circ_0000567. In addition, we found that circ_0000567 could be amplified much better when using a random primer during reverse transcription compared to when using the oligo(dT) primer. However, for the linear *SETD3* transcript, there was no difference when using the oligo(dT) primer or the random primer (**Figure 5B**). This is because the linear transcript has a 3' poly(A) tail, unlike circ RNA. Subsequently, we measured the expression of circ_0000567 in LUAD cell lines and 73 LUAD specimens using qRT-PCR. circ_0000567 showed low expression in A549, H1299, and PC9 cell lines compared to that in the BEAS/2B cell line (**Figure 5C**), and circ_0000567 expression levels were significantly reduced in LUAD specimens compared to that in paired normal lung tissues (**Figure 5D**), which was completely opposite to miR-421.

To study the function of circ_0000567 in LUAD, we overexpressed it in the LUAD cells. The dramatic upregulation of circ_0000567 expression in A549 cells showed that circ_0000567 was successfully overexpressed by the circ_0000567-pLCDH-ciR plasmid (**Figure 5E**). The transwell assay showed that circ_0000567 overexpression significantly decreased the migratory and invasive abilities of A549 cells (**Figure 5F-H**).

Furthermore, the rescue experiment with the transwell assay verified thatoverexpression of circ_0000567 in A549 cell lines significantly inhibited LUAD migration and invasion, and this effect was neutralized after the cells were co-transfected with miR-421 mimics (**Figure 5I-K**).

### *TMEM100* is directly regulated by miR-421 in LUAD

**Figure 6:**
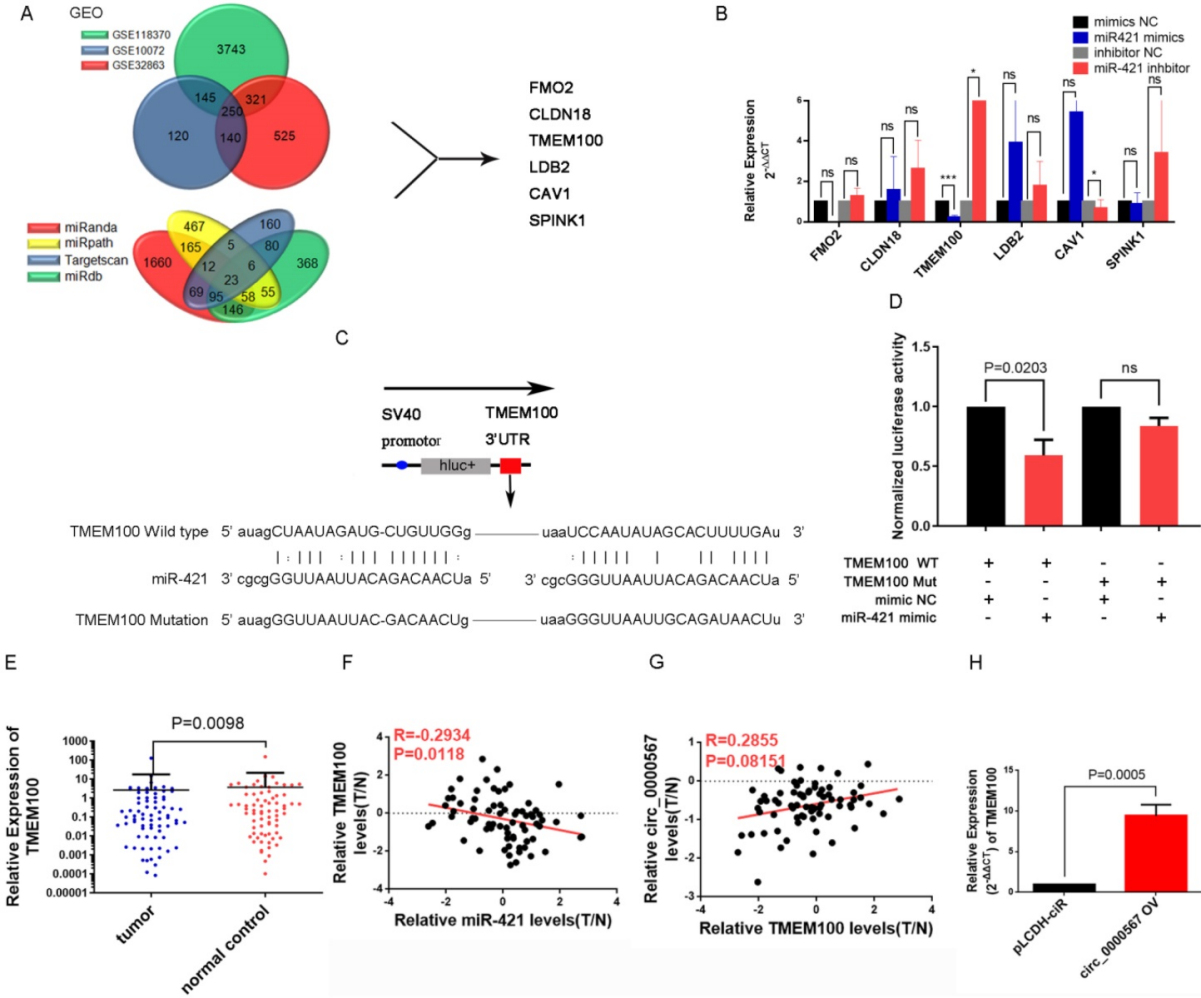
miR-421 promoted migration and invasion of lung adenocarcinoma (LUAD) by the circ_0000567/miR-421/*TMEM100* competing endogenous RNA network. **A** Schematic of the screening procedure of candidate genes. *FMO2*, *CLDN18*, *TMEM100*, *LDB2*, *CAV1*, and *SPINK1* were predicted using TargetScan, miRanda, MirDB, or miRpath websites, and were downregulated in LUAD patients from the Gene Expression Omnibus database. **B** *TMEM100* was upregulated when A549 cells were transfected with miR-421 inhibitor and downregulated when PC9 cells were transfected with miR-421 mimics. *P<0.05, ***P<0.001. The expression of *FMO2*, *CLDN18*, *LDB2*, *CAV1*, and *SPINK1* did not change significantly in PC9 and A549 cells. **C** Schematic of *TMEM100* wild-type (WT) and mutant luciferase reporter vectors. **D** Luciferase activities of WT and mutant *TMEM100* after co-transfection with miR-421 mimics. **E** *TMEM100* was downregulated in 73 LUAD. specimens compared to the paired normal lung tissues (P=0.0098). **F** The expression of miR-421 was inversely proportional to that of *TMEM100* in 73 LUAD tissues. **G** The expression of *TMEM100* was directly proportional to circ_0000567 in 73 LUAD tissues. **H** The expression of *TMEM100* was upregulated when circ_0000567 was overexpressed in A549 cell lines.

According to the typical ceRNA mechanism, the target gene of miR-421 should be a suppressed gene and would be downregulated in LUAD tissues. To identify the target gene of miR-421, we used the LUAD microarray datasets GSE10072, GSE32863, and GSE118370 from the Gene Expression Omnibus (GEO) database (http://www.ncbi.nlm.nih.gov/geo/) to filter differentially expressed genes. Bioinformatics websites, including TargetScan, miRanda, MirDB, and miRpath, were also used to predict potential target genes and their corresponding binding sites. Finally, we identified six genes (*FMO2*, *CLDN18*, *TMEM100*, *LDB2*, *CAV1*, *SPINK1*) that were downregulated in all three GEO searches; they were also predicted as target genes by the bioinformatics websites (**Figure 6A**). Next, we detected changes in the expression levels of these six genes in PC9 and A549 cell lines when transfected with miR-421 mimics and inhibitors, respectively. As illustrated in **Figure 6B**, the expression level of *TMEM100* was downregulated when miR-421 was overexpressed by miR-421 mimics and upregulated when miR-421 was blocked by miR-421 inhibitors. However, *FMO2*, *CLDN18*, *LDB2*, *CAV1*, and *SPINK1* did not have these features in the LUAD cell lines (**Figure 6B**). To verify the sponging relationship between *TMEM100* and miR-421, we constructed wild-type and mutant* TMEM100* luciferase reporters. The pmiR-RB-REPORT™ vector and the binding site (mutant site) are shown in **Figure 6C**. PC9 cells were co-transfected with wild-type or mutant *TMEM100* luciferase reporters and miR-421 mimics or mimic NC. As illustrated in **Figure 6D**, after 48 h of transfection, miR-421 notably impeded the luciferase activity of the wild-type *TMEM100* reporter, whereas it had no such influence on the luciferase activity of the mutant *TMEM100* reporter. Furthermore, the expression of *TMEM100* was confirmed to be downregulated in LUAD tissues compared to that in paired normal lung tissues (**Figure 6E**). In addition, qRT-PCR analyses showed that the expression of miR-421 was inversely proportional to that of *TMEM100* in the 73 LUAD tissues (**Figure 6F**). As for the relationship between circ_0000567 and *TMEM100*, we found that the expression of circ_0000567 was directly proportional to that of *TMEM100* in all 73 LUAD tissues (**Figure 6G**). Furthermore, *TMEM100* was upregulated when circ_0000567 was overexpressed in A549 cells (**Figure 6H**). These findings verified that *TMEM100* was the target gene of miR-421 in LUAD; therefore, it could also be regulated by a miR-421 sponge, namely circ_0000567.

In summary, miR-421 was an independent predictor of shorter OS in patients with LUAD. circ_0000567 could sponge miR-421, releasing the target gene of miR-421 (*TMEM100*), thus regulating the invasion and migration of LUAD. We suggest that miR-421 promotes LUAD migration and invasion via the circ_0000567/miR-421/*TMEM100* axis.

## Discussion

Although the diagnosis and treatment of LUAD are constantly improving, LUAD is a high-risk disease with a 5-year survival rate of 21%, and the potential mechanism underlying the development and progression of LUAD is unclear. However, it is becoming increasingly clear that ncRNAs play a crucial role in cancer development and progression and can affect the hallmarks of cancer. Our finding of the OS-related ncRNA, miR-421, holds great promise for the improvement of prognostic prediction and therapeutic processes in LUAD. Furthermore, studying the mechanism of the circ_0000567/miR-421/*TMEM100* axis in LUAD helps us deepen our understanding of the regulation of ncRNAs, and is expected to identify new targets for LUAD.

miRNAs are small, single-stranded, evolutionarily conserved ncRNAs. Interestingly, they mainly regulate gene expression at the post-transcriptional and translational levels by targeting the 3′-UTRs of mRNAs at their binding sites 34-36. Nadal et al. found that the miRNA expression profiles of different morphological subtypes of LUAD were very distinct 37. Previous studies have found that some miRNA panels are associated with prognosis in LUAD. For instance, Xin et al. suggested the usefulness of an miRNA profile including let-7i, mir-1976, mir-199a-1, mir-31, mir-3940, mir-450a-2, mir-4677, mir-548v, and mir-6803, which could predict survival in patients with LUAD 32. In our study, a panel of six miRNAs, including miR-3940, miR-873, miR-550a-2, miR-1293, miR-421, and miR-212, was predicted to be associated with shorter OS of patients with LUAD according to Cox and Kaplan-Meier analyses of 240 patients from TCGA, and the impact of these six miRNAs needs to be further verified by our experiments.

Notably, most of the miRNAs screened in the previous literature were selected through differentially expressed miRNAs in different populations, paired tumor and normal tissues, or through expression stability, using gene microarrays, next-generation sequencing, and bioinformatics prediction methods 38-44. However, in our study, we screened target miRNAs according to their HR in predicting the prognosis of LUAD patients. We selected miR-421 for further analyses because of its high hazard ratio in predicting poor OS and its relatively high expression level in LUAD, as determined using RNA-seq data and corresponding clinical data downloaded from TCGA. Taken together, miR-421 is a prognostic biomarker for LUAD.

In previous studies, miRNAs were regarded as diagnostic or prognostic markers, as well as related to driver genes and therapeutically targeted molecules in LUAD 45. It has been reported that miR-374a and miR-374b are associated with poor prognosis; miR-224, miR-147b, and miR-31 are related to lymph node metastasis and prognosis in LUAD 46. Recently, miR-421 was found to be dysregulated in breast and colon tumors 47, 48.It has been reported that miR-421 promotes the development of osteosarcoma by regulating the target gene *MCPIP1* and that it might serve as a valuable biomarker in patients with esophageal adenocarcinoma and osteosarcoma 49-51. Chen et al. found that overexpression of plasma miR-421 can act as a novel biomarker for the detection of precancerous lesions and early gastric cancer 52. Most importantly, miR-421 was reported to be overexpressed in NSCLC, and overexpression of miR-421 might serve as a prognostic biomarker 53, 54. Nevertheless, the biological function and molecular mechanism of miR-421 in LUAD require further research. Our results illustrate that miR-421 is highly expressed in LUAD cell lines and specimens, miR-421 promotes the migration and invasion of LUAD cells significantly, and patients with high miR-421 expression have a shorter 5-year survival. These findings provide robust evidence that miR-421 plays a significant oncogenic role in LUAD, which needs to be further studied.

circRNAs are a newly defined family of ncRNAs, consisting of a covalently closed loop structure without 5ʹ-3ʹ polarity or a poly(A) tail, preventing degradation by RNA exonucleases. The ceRNA hypothesis suggests that RNA transcripts of circRNAs can compete with mRNAs via miRNAs. They usually bind to miRNAs and regulate the expression of miRNA target gene transcripts that harbor the same miRNA-binding sites, constructing a complex post-transcriptional regulatory network. According to Peng et al., 11 upregulated circRNAs and 2 downregulated circRNAs were identified in LUAD 55. These circRNAs have been demonstrated to be associated with the migration, invasion, apoptosis, and other aspects of tumor progression, and they serve as biomarkers for the diagnosis or prognosis of LUAD 20-31. In our study,we found that miR-421 could be sponged by the circRNA circ_0000567, so that miR-421 could not bind to and inactivate the target gene *TMEM100*. circRNAs can be divided into three major subtypes based on their mechanism of formation: exon circRNAs (ecircRNAs), exon-intron circRNAs (elciRNAs), and circular intronic RNAs (ciRNAs). circ_0000567 is an ecircRNA spliced from *SETD3* and has already been demonstrated to be a potential diagnostic biomarker in colorectal cancer; it has also been found to sponge miR-421 to inhibit hepatocellular carcinoma (HCC) growth 56. In the current study, circ_0000567 was lowly expressed in LUAD cells and specimens, and could suppress migration and invasion in A549 cells, while miR-421 has the opposite effect and could partly counteract the effect of circ_0000567. A luciferase assay confirmed the binding site between miR-421 and circ_0000567. The negative correlation between the expression levels of miR-421 and circ_0000567 also proved their relationships. In summary, circ_0000567 acts as an RNA sponge to bind miR-421 and affect its function. Our finding of circ_0000567 as a miR-421 sponge is the first to suggest the important role of this circRNA in LUAD, which enriches the circRNA expression profile of LUAD, and suggests and verifies the suppressor role of circ_0000567 in LUAD.

As for the downstream target, we suggest that miR-421 promotes the migration and invasion of LUAD cell lines by targeting *TMEM100* mRNA in LUAD. In 2012, Somekawa S et al. first suggested that TMEM100 may play indispensable roles during endothelial differentiation and vascular morphogenesis via BMP9/BMP10/ALK1*/TMEM100* signaling. *TMEM100* is also known as a tumor-suppressing gene that is downregulated and differentially expressed in cancers. For instance, it has been reported that* TMEM100* functions as a tumor suppressor in HCC metastasis and proliferation 57. Recently, another study demonstrated that *TMEM100* functions as a cancer suppressor mainly by inhibiting the TNF signaling pathway in NSCLC 35, which is consistent with our results. In our study, *TMEM100* was predicted as a target gene for miR-421 using bioinformatics analyses according to the GEO database and miRanda. *TMEM100* was expressed at low levels in LUAD specimens and could be downregulated and upregulated by miR-421 mimics and inhibitors in A549 and PC9 cells, respectively. *TMEM100* could also be upregulated by circ_0000567 in LUAD cells. A luciferase assay confirmed the binding sites. Most importantly, we found that *TMEM100* expression was inversely proportional to miR-421, but directly proportional to circ_0000567 in 73 LUAD specimens obtained from patients in our department. Taken together, these results support the hypothesis that miR-421 promotes LUAD progression via a ceRNA mechanism and is sponged by circ_0000567, which results in the loss of the ability to inactivate the downstream target gene *TMEM100*.

In summary, we observed that the expression level of miR-421 was significantly correlated with shorter OS in 240 LUAD patients from the TCGA database. Moreover, this result was verified in 73 LUAD specimens obtained from our department. In an *in vitro* experiment, we found that enhancement of miR-421 promoted cell migration and invasion, while inhibition of miR-421 had the opposite effect. circ_0000567, which was predicted from three bioinformatics websites, binds to miR-421 and subsequently inhibits its suppressive capabilities on the target gene TMEM100. miR-421 serves as a novel oncogenic miRNA and promotes migration and invasion via the circ_0000567/miR-421/*TMEM100* axis in LUAD. Most importantly, miR-421 may be a potential prognostic marker in patients with LUAD, pointing to novel directions for prognostic prediction and treatment of LUAD.

## Supplementary Material

Supplementary figures and table.

## Figures and Tables

**Table 1:** MicroRNAs related to overall survival ranked by hazard ratio

No.	Name	EXP (coef)	P	Mean expression	No.	Name	EXP (coef)	P	Mean expression
1	hsa-mir-1292	1.6323	0.0096	0.5301	32	hsa-mir-450b	1.0104	0.0260	19.0439
2	hsa-mir-1254	1.2914	0.0198	0.9335	33	hsa-mir-449b	1.0079	0.0454	2.7930
3	hsa-mir-3940	1.2859	0.0341	1.0549	34	hsa-mir-330	1.0075	0.0257	48.1913
4	hsa-mir-3682	1.2591	0.0068	1.1837	35	hsa-mir-31	1.0052	0.0000	22.9920
5	hsa-mir-3691	1.2471	0.0303	0.7416	36	hsa-mir-128-2	1.0039	0.0485	73.5355
6	hsa-mir-320d-1	1.2352	0.0493	0.5504	37	hsa-mir-222	1.0038	0.0112	83.3141
7	hsa-mir-1262	1.1545	0.0038	1.2262	38	hsa-mir-589	1.0034	0.0094	109.7946
8	hsa-mir-873	1.1318	0.0000	0.6873	39	hsa-mir-1912	1.0029	0.0292	3.3274
9	hsa-mir-7-2	1.1203	0.0020	1.0004	40	hsa-mir-193b	1.0028	0.0334	78.3203
10	hsa-mir-7-3	1.1067	0.0082	0.9771	41	hsa-mir-424	1.0025	0.0002	89.8590
11	hsa-mir-550a-2	1.0938	0.0006	3.3696	42	hsa-mir-193a	1.0021	0.0289	210.0761
12	hsa-mir-550a-1	1.0819	0.0008	4.1603	43	hsa-mir-584	1.0020	0.0425	40.7900
13	hsa-mir-1293	1.0554	0.0386	0.9611	44	hsa-mir-1298	1.0010	0.0297	9.4789
14	hsa-mir-450a-2	1.0470	0.0067	4.6204	45	hsa-mir-542	1.0009	0.0139	293.2192
15	hsa-mir-147b	1.0440	0.0127	3.1276	46	hsa-mir-1911	1.0005	0.0301	21.6588
16	hsa-mir-421	1.0430	0.0016	4.7425	47	hsa-mir-582	1.0004	0.0001	412.0279
17	hsa-mir-760	1.0425	0.0001	3.2995	48	hsa-mir-27a	1.0004	0.0069	1517.0722
18	hsa-mir-450a-1	1.0390	0.0340	4.6926	49	hsa-mir-24-2	1.0003	0.0142	2119.4670
19	hsa-mir-3677	1.0335	0.0065	7.3630	50	hsa-mir-192	1.0000	0.0290	2348.2164
20	hsa-mir-212	1.0292	0.0484	9.8931	51	hsa-mir-10b	1.0000	0.0002	7288.5585
21	hsa-mir-95	1.0282	0.0062	4.4191	52	hsa-mir-100	1.0000	0.0233	8905.1132
22	hsa-mir-138-1	1.0253	0.0496	3.3045	53	hsa-mir-30a	1.0000	0.0235	18419.6048
23	hsa-mir-935	1.0223	0.0001	3.3771	54	hsa-mir-338	0.9995	0.0270	769.6756
24	hsa-mir-412	1.0165	0.0467	4.0426	55	hsa-mir-30c-2	0.9990	0.0430	458.2823
25	hsa-mir-365-2	1.0151	0.0070	19.6787	56	hsa-mir-3065	0.9976	0.0245	178.0391
26	hsa-mir-769	1.0146	0.0413	23.4524	57	hsa-mir-181c	0.9944	0.0170	116.9814
27	hsa-mir-365-1	1.0131	0.0153	20.0419	58	hsa-mir-660	0.9906	0.0407	47.8742
28	hsa-mir-340	1.0124	0.0364	29.2571	59	hsa-mir-1287	0.9905	0.0049	64.1908
29	hsa-mir-7-1	1.0111	0.0220	23.9518	60	hsa-mir-1468	0.8968	0.0340	4.2465
30	hsa-mir-505	1.0111	0.0055	41.9137	61	hsa-mir-3189	0.7249	0.0317	1.0393
31	hsa-mir-33a	1.0105	0.0025	22.4100					

**Table 2:** Correlations between miR-421 expression and clinical characteristics in lung adenocarcinoma patients (n=73)

Variable	No. of cases	miR-421 expression		P
		low	high	
Total	73	37	36	
Age (years)				
≤60	44	23	21	0.7382
>60	29	14	15	
Sex				
Male	36	14	22	**0.0468**
Female	37	23	14	
Smoking				
No	43	26	17	**0.0454**
Yes	30	11	19	
TNM stage				
I/II	53	29	24	0.2620
III	20	8	12	
Differentiation				
Poor/moderate	67	31	36	**0.012**
Good	6	6	0	
5-year survival				
No	26	8	18	**0.0114**
Yes	47	29	18	

**Table 3:** Multivariate overall survival analysis of prognostic factors for lung adenocarcinoma patients (n=73)

Variable	Overall survival		
	HR	95% CI	P
Age (≤60 vs. >60 years)	-	-	0.140
Sex (male vs. female)	-	-	0.086
Smoking (no vs. yes)	-	-	0.928
Pathological stage (I/II vs. III)	-	-	0.090
Differentiation (poor/moderate vs. good)	-	-	0.714
miR-421 expression (low vs. high)	2.593	1.127-5.966	0.025

CI, confidence interval; HR, hazard ratio
