# Supplementary material for: circ_0000567/miR-421/TMEM100 Axis Promotes the Migration and Invasion of Lung Adenocarcinoma and Is Associated with Prognosis
Source: J Cancer. 2022 Feb 28;13(5):1540–52. doi: 10.7150/jca.60124 (PMC8965118; doi:10.7150/jca.60124)

Supplementary Materials

Table S1 Primers

Name	Primers (5’→3’)
**U6**	Forward CTCGCTTCGGCAGCACA
	Reverse AACGCTTCACGAATTTGCGT
**Hsa-miR-421**	Forward ATCAACAGACATTAATTGGGCGC
	Reverse GCGAGCACAGAATTAATACGAC
**GAPDH**	Forward ACTCCTCCACCTTTGACGC
	Reverse GCTGTAGCCAAATTCGTTGTC
**Circ_0000567**	Forward TGAAGAAGATGAAGTTCGGTAT
	Reverse GTGCCAGATTTCTGAGTTTT
**β-actin**	Forward AGAGCTACGAGCTGCCTGAC
	Reverse AGCACTGTGTTGGCGTACAG
**TMEM100**	Forward CATGGCAGCGACGATGGAGAAG
	Reverse CCGGCGATGAAGACAACCACAG
**hsa-miR-873-5p**	Forward GCAGGAACTTGTGAGTCTCCT
	Reverse GCGAGCACAGAATTAATACGAC
**hsa-miR-3940-3p**	Forward CAGCCCGGATCCCAGCCCACTT
	Reverse GCGAGCACAGAATTAATACGAC
**hsa-miR-550a-3p**	Forward TGTCTTACTCCCTCAGGCACAT
	Reverse GCGAGCACAGAATTAATACGAC
**hsa-miR-1293**	Forward TGGGTGGTCTGGAGATTTGTGC
	Reverse GCGAGCACAGAATTAATACGAC
**hsa-miR-212**	Forward ACCTTGGCTCTAGACTGCTTACT
	Reverse GCGAGCACAGAATTAATACGAC

Figure S1 K-M analyses using the KM plotter website (http://kmplot.com/analy sis/) of 504 LUAD patients. **A-F** Patients with higher expression levels of miR-3940, miR-873, miR-550a-2, miR-1293, miR-421 and miR-212 had a shorter OS than those with lower expression levels of these miRNAs.

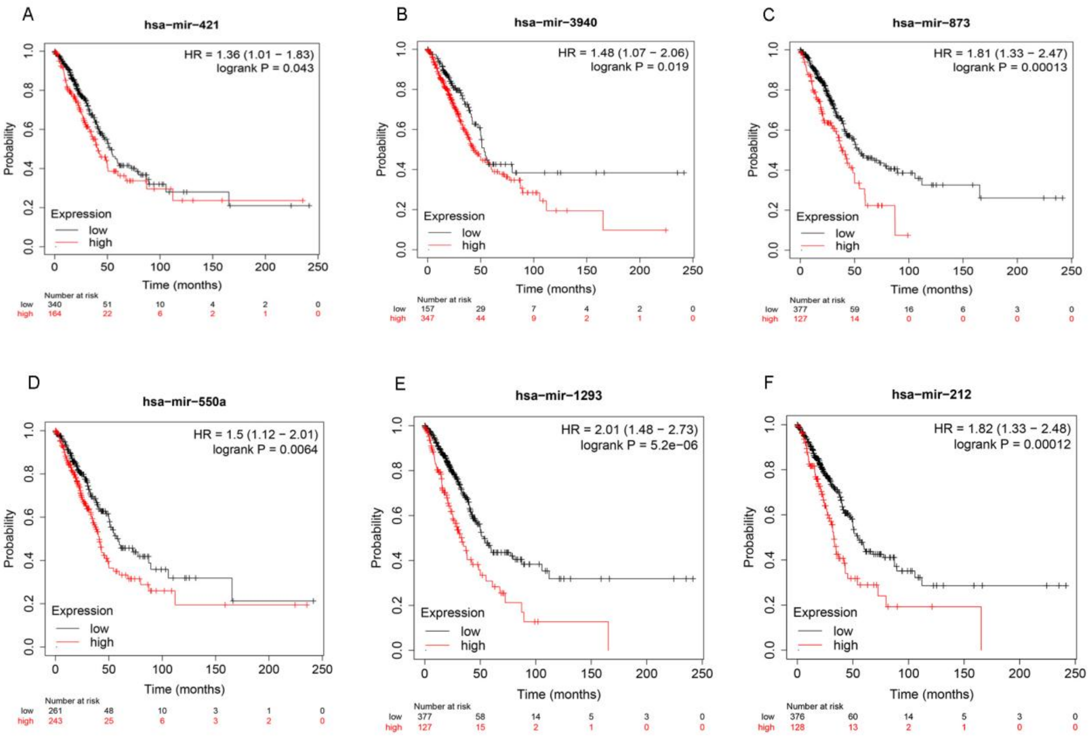

Figure S2 The relative expression levels of 6 miRNAs according to the TCGA datasets.

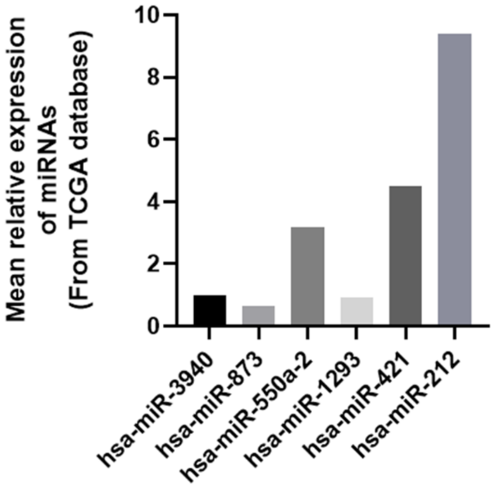

Supplement: Supplementary file 1 — Supplementary figures and table. [file jcav13p1540s1.pdf]
